# THE MULTIPLE DIMENSIONS OF THE RACE EXPERIENCE AND ASSOCIATIONS WITH HEALTH IN OLDER ADULTS

**DOI:** 10.1093/geroni/igz038.2969

**Published:** 2019-11-08

**Authors:** Jerry Kaufman

**Affiliations:** University of Chicago, Chicago, Illinois, United States

## Abstract

Race is experienced along a number of dimensions. In the United States, education, family background (e.g., parents’ education), skin shade, experiences of racial discrimination, neighborhood racial composition, state/region of birth, and interracial marriage help to define the “race experience.” Many of these factors have been individually associated with adverse outcomes for African Americans relative to Whites, but little research has examined how these factors cohere within individuals. Using a national survey of African American and White older adults, we employed latent class analysis and, in preliminary analyses, identified three clusters of individuals who were characterized by unique race experiences. We then assessed and determined that these clusters were also unique in their differential associations with health outcomes. This data-driven approach will provide insight into the profiles of individuals whose race experience contributes to health inequities among older Americans.

